# The impact of health on labour outcomes among middle-aged and elderly: Insights from India

**DOI:** 10.1371/journal.pone.0303194

**Published:** 2024-07-11

**Authors:** Umenthala Srikanth Reddy, K. S. James

**Affiliations:** 1 International Institute for Population Sciences (IIPS), Mumbai, India; 2 One Health Trust, Bengaluru, Karnataka, India; 3 Newcomb Institute, Tulane University, New Orleans, LA, United States of America; India Health Action Trust (IHAT), Uttar Pradesh Technical Support Unit (UP-TSU), INDIA

## Abstract

The impact of ill-health on labour force participation is a well-recognized concern in both developed and developing countries. However, previous studies have often overlooked age differentials in this relationship, assuming uniform effects across age groups. This study aims to fill this gap by examining how ill-health affects labour outcomes among different age segments in India. Utilizing data from the Longitudinal Ageing Study in India (LASI) Wave 1, which covers over 72,000 individuals aged 45 and above, this research investigates the linkage between health and labour force outcomes. The labour outcomes in this study includes labour force participation, labour earnings and hours worked. Present study used instrumental variable approach to mitigate endogeneity issues and establish causal relationships between health and labour outcomes. The Heckman selection model is utilized to address selection bias in analysing wage and hours worked. The study reveals several key findings. Firstly, ill-health consistently leads to a decline in labour force participation among both middle-aged (28 percent) and elderly (36 percent) individuals in India. This underscores the pervasive impact of health on workforce engagement, particularly in a context with limited social security measures. Secondly, the research identifies significant variations in the effects of ill-health on wages and hours worked based on age. Among elderly individuals, there is a pronounced reduction in both wages and hours worked due to ill-health. However, this effect is less pronounced among middle-aged adults. Furthermore, socioeconomic factors play a pivotal role in shaping how ill-health influences labour outcomes among different age groups. This study underscores the importance of considering age differentials in the impact of ill-health on labour outcomes, offering valuable insights for policymakers, practitioners, and researchers seeking to address this critical issue in India’s dynamic socio-economic landscape.

## Introduction

It is well recognized that health plays an important role in labour outcomes. In line with Grossman’s (1972) [1] research, good health is presumed to improve productivity and the labor supply decisions of the individual. Numerous studies have demonstrated the negative effect of ill-health on labour market participation across countries including developing countries like India. However, these studies do not provide an answer on the impact of this relationship at different ages particularly among the middle and elderly population. In other words, the impact of ill-health on the labour outcomes is assumed to be uniform across different age segments by various studies and as such misses an important aspects of age in mediating such a relationship [2–4].

India, in recent times, has witnessed rapid demographic and epidemiological changes [5]. With the demographic changes and the consequent age structure transition, the age distribution of the population has substantially changed with increasing proportion of elder persons across countries. The work participation, too, of elder persons is relatively common due to the informal nature of work as well as due to a lack of social security system. It is also found that the onset of disease in India is typically a decade earlier (> = 45 years of age) than in developed countries (55 years) [6], which may result in people living longer with extended years of ill-health, impacting their labour outcomes. These changes are likely to affect the labour outcomes differently among different age groups. They have the potential to jeopardise the household economy. However, none of the available studies have considered impact of age differentials when understanding the nexus between health and labour force participation.

The negative effects of ill-health at later ages are addressed through pension, social security and retirement schemes in developed countries. However, in countries like India even a transitory ill-health may have severe consequences. Ill-health might result in complete withdrawal of labour or a reduced labour productivity [7]. It also results in economic consequences such as asset selling or informal borrowings. Ultimately, the poor may fall back on one asset they have, which is labour [8]. Ill-health consequences may be severe among poor households due to limited access to insurance [9] and no formal mechanism of unemployment benefits [10]. All these consequences are likely to vary significantly across different age segments. A middle age adult may be the main breadwinner for the family, while an elderly most likely to be in labour force as coping mechanism to avoid risk of falling into poverty. Thus, understanding health and the consequent labour outcomes in different age segments is important for designing welfare measures.

Ill-health does have a negative effect on labour outcomes and the magnitude of the effect can exhibit variation across age groups and regions. For example, studies in Australia by Cai and Klab [11] and in Sri Lanka by Sisira and Samaratunge [2] found that elderly are more likely to withdraw from labour force participation when suffering from a disease or due to an ill-health condition. But in United Kingdom [4], a study found no difference in the effect of health on labour force participation between young and elderly. In Russia for the urban sample, with the increase in age the relationship between working status and poor health was getting stronger until retirement age and then it got weakened thereafter [12]. Thus, the stark difference across age segments in different countries suggests the wide variation in the public policies existing in these countries. Strictly, the evidence from one country may not be relevant to other countries, particularly among the older ages due to variations in health and labour policies across countries.

In this study, we examine the relationship of ill-health on the labour force participation separately for different age segments (Middle aged 45–59 and Elderly 60+). It is postulated that the mechanisam of this relationship would be differnet across age groups and a universal finding may not be very relevant from a public policy point of view. The existing studies have often found that when people’s health is not good, they tend to work less (eg: [3, 13–16]). This finding, however, may be more relevant for mildde adult and elderly population. It is likely that this relationship may be more adverse for individuals in older ages with more of a possibility of withdrawal from the labour market than mere less work. The existing studies only provide conflicting result on the relationship between ill-health and work participation of older persons. For instance, Dureja and Negi [15], suggests that when people are sick, they tend to earn less money and work fewer hours especially as they get older. As against this, Ghatak & Madheswaran [17], found that long-term illness might not always mean earning less money.

Not withstanding the fact that ill-health will either lead to less particiation in labour market or complete withdrawal, it is also important to assess the extent of decline in the participation due to ill-health from a policy point of view. The available studies in India merely state the direction of this relationship (see e.g, [3, 13–16]. But, we still don’t know exactly how many hours of work is sacrificed due to ill-health and the extent of wage loss at different ages. Generally studies also observed that ill-health has a declining effect on wage in India [15] although it has not quantified the extent of wage deprivation. Contrary to this study, Ghatak & Madheswaran (2011) [17] found that chronic illness has no effect on wages while BMI has a positive effect with respect to age and wages but only upto a certain age and then has a declining effect. Given these conflicting research result, it is important to quantify the effect particularly across different ages.

In this paper we try to ask two questions relating to the relationship between compromised health and its labour outcomes. First, whether the relationship between ill-health and labour outcomes remain same across ages in India. Specifically, the study tries to identify the causal relationship between health and labour force activity across middle aged and elderly in India. We also examine in which age group, the withdrawal from the labour activity is the highest. Secondly, the study tries to answer, when conditioned on the work status of individuals, how ill-health will affect wage earning and hours worked across age groups.

## Data and methods

For this study we have used Longitudinal Ageing Study in India, Wave 1. LASI is a full-scale national survey of scientific investigation of the health, economic, and social determinants and consequences of population aging in India, conducted in 2017–18. It is a nationally representative cross sectional survey of over 72,000 older adults aged 45 and above across India’s states and union territories.

The LASI data is used to test how health affects labour force participation in India. For constructing the labour force participation variable, we have included the people who are currently working and also the people who are in search of a job. Chronic self-reported diseases, difficulty in mobility and Activity Daily Living (ADL) are used as instrumental variables for general health status. Self-reported health (Excellent, Very Good, Good, Fair and Poor) is used as a subjective health indictor. Their effect on labour force participation are assessed using simultaneous equation model. Each instrumental variable is defined as follows.

Chronic self-reported disease: In LASI, questions were asked about ever being diagnosed with disease. The one who reported being diagnosed with diseases like Blood Pressure, Diabetes, Cancer, Chronic Heart Disease, Stroke, Arthritis, Neurological Disorders, and High Cholesterol were considered as suffering from an illness. Person who reported any one disease has been considered as suffering from ill-health.

Activity of Daily Living: For Activity of Daily living we have considered person who couldn’t perform or has difficulty in performing activities likes dressing, walking across a room, eating, getting in or out of bed, using toilet, preparing meals, shopping, making calls, taking medication, manging bills, finding address. A score was generated by summing up all the difficulities faced by individuals. The score has been recoded into binary variable as person who scored more than oneADL has considered as suffering from ADL and who scored zero as not suffering from ADL.

Difficulty in mobility: Apart from ADL and disease status we have included one more instrument variable for the analysis. In this variable we have considered having difficulty if person has reported any of the following difficulties either due to health or physical problems. The difficulties included are Walking 100 yards, sitting for more than 2 hours, difficulty in getting up from a chair, climbing on stairs, and stooping, kneeling, or crouching, reaching or extending arms above shoulder level, carrying of larger objectives, picking up a coin. Persons reporting any of the difficulties were considered as having difficulty due to physical or due to health reasons.

Labour force: Person who reported that they are currently working and looking for work are considered to be in labour force.

Apart from the above mentioned variables we have considered other control variables like gender, education, wealth of the household, caste and religion,spouse employment, marital status, physical activity, ever smoked, ever consumed alcohol, MPCE quintile and presence of children in household‥

### Empirical specification

As discussed, in estimation of the effect of health on labour force participation there arises endogeneity problem of health variable. To mitigate the endogeneity problem we need to find a variable known as “instrument” that is correlated with health but not with labour force participation. In this analysis we first discuss the effect of health on labour force participation using ordinary least square regression (OLS) with different model specification. Using OLS regression we have five specifications, the first specification is a null model representing a correlation between health and labour force, then adding individual level fixed effects in second specification, then state level fixed effects in third, PSU level fixed effects in the last specification.

However, using OLS regression can be problematic given the nature of health variable being endogenous. Thus we used an instrumental variable strategy, which is described as below.

In order to overcome biased regression coefficient problems from OLS, we adopted Stern (1989) [18] and Cai (2010) to estimate the effect of health condition on labour force participation. We used simultaneous equation model to address the endogeneity of health, apart from measure of health as self-reported we included chronic illness, difficulty in mobility and ADL measures. In this estimation strategy the first stage equation represents a self-reported health as function of a set of exogenous variables including instrumental variable of health and then predicts the probability of health. The predicted probabilities will be used as a proxy for health variable in the second equation that is labour force participation decision.

In the frameowrk used by Stern (1989) and Cai (2010), they addressed a question of variation in labour force participation by accounting endogeneity of health. The first equation in this model describes the determinants of health as follows

$$\text{Self-reported}\;\text{health}\left(\text{h}**\right)=\gamma 1\text{h}*+\text{xh}*\beta +\varepsilon 1$$ (1)

In the Eq (1) h** is Self-reported health, which will depend on the instrumental health variable h* and xh is a set of exogenous variables that determine health, ε1 is error term. As the first equation, which represents the self-reported health, depends on instrumental variable and individual characteristics (exogenous variables).

In second we specify the effect of latent health on labour force participation as follows

$$\text{LFPR}\left(\text{p}*\text{t}\right)=\gamma 2\left(\text{h}**\right))+\varphi \text{l}\left(\text{exogenous}\;\text{variables}\right)+\varepsilon 2$$ (2)

In the above equation p*t represents propensity to participate in labour force, the parameter γ2 represents the effect of health on labour force participation. In the second equation the propensity to be in labour force and to be out of labour force is a function of latent self-reported health and set of exogenous variables.

We used Wald test to know whether a variable is endogenous or not. The reported test statistic and p-value have been reported at the end of each model and for each instrument used. If there is no endogeneity found then a standard regression model will be more appropriate to estimate the effect of health on labour force participation. In our study we found no such evidence to reject health as an exogenous variable. In each specification we found that health is an endogenous variable. So Instrumental regression deemed to be appropriate to study the causal effect of health on labour force participation.

### Heckman selection model

In the second stage of our analysis, we used Heckman selection model to know the effect of health on worked hours and earnings. In this model we have two equations, the first equation describes ability to participate in labour force and in the second if participated then what are the number of hours spent working and the amount of money earned There is a selection bias when analysing wage and hours worked is used since those with positive hours worked and wages are not randomly observed from population resulting in a selection bias. Let us assume yw* denote the outcome of interest, here log of hours worked and log of monthly wage earned. Here we introduce a latent variable yp and outcome variable yw* is observed only when yp>0. Here yp determines if probability of work participation, yw* determines the level of hours working and income earned through participation.

Heckman model assumes that there exist an underlying relationship defined as

$$\text{yw}*=\beta *{\text{x}}_{\text{j}}+{\text{u}}_{\text{ij}}$$ (3)

here yw* is only observed when

yp*λ+u2j>0 (4)

When the correlation between u1 and u2 is not equal to zero then standard regression techniques on wage and hours worked equation will lead to biased results. In each of the reported results in our estimation, the correlation coefficients between these equations is greater than zero thus suggesting a selection bias.

Eq (4) represents wages of respondents, whereas Eq (5) is probit model for all the respondents that is for those who are in and out of labour force. From both these equation inverse mills ratio is calculated, the ratio calculated from the estimated probit model is considered as an adjustment factor for sample selection bias with Eq 4 regression. After adding the mills ratio the Eq 4 has been converted as follows

yw*=xjβ+λ*zuij (5)

## Results

### Descriptive statistics

Table 1 provides percentages of population working in different age groups by various measures of ill-health status. At all India level, the working status of individuals aged greater than 45 years is about 68.22 percent. As age advances, we observe a decline in work participation, however, 56 percent of the elderly in the age group 61–70 years and 29 percent of the elderly aged above 71 years are currently working. International comparison especially among elderly shows that labour force participation among elderly in India is higher than those in developed countries and some developing countries such as Brazil and South Africa [19]. With age, suffering from chronic diseases, difficulty in mobility and difficulty in activity of daily living has increased. Table 1 represents working status, ADL, presence of any chronic disease and difficulty in mobility by various socioeconomic backgrounds. Overall we found that 70 percent of the respondents are engaged in labour force activity, 55 percent are suffering some kind of chronic morbidity, 61 percent are suffering from difficulty in movements and 40 percent of persons have difficulty in performing daily activities. Among social groups, schedule caste and tribe have reported higher working status and suffering from any chronic morbidity as compared to OBC and others. The poorest quintile have suffered higher from chronic morbidity, difficulty in walking and ADL.

**Table 1:** "Percentage of individuals currently working, suffering from chronic diseases, experiencing mobility difficulties, and ADL challenges by age".

Age	Working Status	Suffering from Chronic Disease	Difficulty in Mobility	Activity of Daily Living	Sample (N)
45–50	90.22	45.62	44.76	24.65	16,288
51–60	82.74	55.7	59.2	34.22	20,934
61–70	56.55	61.99	71.62	45.94	18,076
71+	29.75	65.47	83.8	64.13	10,263
**Total**	**68.22**	**56.97**	**63.51**	**59.59**	**65,561**

Source: Author’s Calculation from LASI data.

### Effect of Ill-health on labour force participation

As indicated by other studies, it is also found here that the relationship between ill-health and work participation is negative (Table 2). With the increase in ill-health there is a decline in labour force participation. This has been confirmed by adopting different methods in Table 2. Since the regression coefficients from OLS may be biased, we have also employed the instrumental regression (IV) models. The magnitude of the decline is more evident in IV regression, that is with the increase in ill-health 34 percent of the people are more likely to leave labour participation. The Wald and Durbin Score test also confirmed that health is an endogenous variable and OLS may not be very appropriate to conclude about this relationship.

**Table 2:** Comparison of ordinary least square and IV regression on effect of health on labor force activity in india.

Labour force Activity	Ordinary Least Square Regression										IV Regression	
	1		2		3		4		5		6	
	Null Model		Individual		State FE		PSU FE		Household FE		IV Estimates	
	Coeff (*β*)	SE	Coeff (*β*)	SE	Coeff (*β*)	SE	Coeff (*β*)	SE	Coeff (*β*)	SE	Coeff (*β*)	SE
**Self-reported health**	-0.158^***^	-0.005	-0.049^***^	-0.004	-0.055^***^	-0.004	-0.058^***^	-0.005	-0.050^***^	-0.008	-0.346^***^	-0.028
**Age at last birthday**			-0.018^***^	0	-0.018^***^	0	-0.018^***^	0	-0.020^***^	-0.001	-0.016^***^	0
**Gender**												
Male®												
Female			-0.149^***^	-0.005	-0.148^***^	-0.005	-0.139^***^	-0.005	-0.106^***^	-0.007	-0.129^***^	-0.006
**Years of Schooling**			-0.004^***^	-0.001	-0.004^***^	0	-0.003^***^	-0.001	0	-0.001	-0.005^***^	-0.001
**Marital Status**												
Others®												
Married			-0.008	-0.006	0.001	-0.006	0.01	-0.006	0.202^***^	-0.019	-0.012	-0.006
**Spouse Employment**			0.072^***^	-0.005	0.066^***^	-0.005	0.044^***^	-0.005	-0.316^***^	-0.01	0.079^***^	-0.005
**Physical Activity**												
No®												
Yes			0.195^***^	-0.005	0.197^***^	-0.005	0.203^***^	-0.005	0.201^***^	-0.009	0.169^***^	-0.006
**Ever Smoked**												
No®												
Yes			-0.001	-0.005	-0.001	-0.005	0.001	-0.005	0.019^*^	-0.009	-0.005	-0.005
**Ever Consumed Alcohol**												
No®												
Yes			-0.005	-0.004	-0.004	-0.004	-0.006	-0.004	0.003	-0.008	-0.025^***^	-0.005
**Residence**												
Rural®												
Urban			-0.027^***^	-0.006	-0.030^***^	-0.006	0	(.)	0	(.)	-0.042^***^	-0.007
**Caste**												
Schedule Tribe®												
Schedule Caste			-0.019^*^	-0.008	-0.034^***^	-0.008	-0.015	-0.01	0	(.)	0.017	-0.009
Other backward class			-0.011	-0.007	-0.032^***^	-0.008	-0.012	-0.009	0	(.)	0.015^*^	-0.008
Others			-0.043^***^	-0.008	-0.047^***^	-0.008	-0.034^***^	-0.01	0	(.)	-0.012	-0.009
**Religion**												
Hindu®												
Muslim			0	-0.008	0.015	-0.008	-0.004	-0.01	0	(.)	0.014	-0.008
Christian			0.030^***^	-0.009	-0.016	-0.01	-0.019	-0.013	0	(.)	0.024^*^	-0.01
Other			0.005	-0.011	-0.002	-0.011	-0.006	-0.013	0	(.)	0.007	-0.011
**MPCE quintile**												
Poorest			0.025^***^	-0.007	0.019^**^	-0.007	0.013	-0.007	0	(.)	0.021^**^	-0.008
Poorer			0.020^**^	-0.006	0.018^**^	-0.006	0.015^*^	-0.007	0	(.)	0.016^*^	-0.007
Middle			0.015^*^	-0.006	0.012^*^	-0.006	0.008	-0.006	0	(.)	0.01	-0.007
Richer			0.019^**^	-0.006	0.019^**^	-0.006	0.018^**^	-0.006	0	(.)	0.018^**^	-0.007
Richest®												
**Children Below 5 years**			-0.013^***^	-0.003	-0.011^***^	-0.003	-0.012^***^	-0.003	0	(.)	-0.014^***^	-0.003
**Children Between 6 and 14 years**			-0.012^***^	-0.002	-0.011^***^	-0.002	-0.011^***^	-0.002	0	(.)	-0.012^***^	-0.002
**First Stage Equation**												
**Activity Daily Living**	-		-	-	-	-	-	-	-	-	0.18***	0.006
**F-statistic for first stage**	-		-	-	-	-	-	-	-	-	795	
**F-Statistic for Second Stage**	914		835		817		791		739		916	
**Observations (N)**	46952		4602		46021		46014		21984		49314	
**Durbin**											186***	
**Wu-Hausman**											187***	

Standardized beta coefficients; Standard errors in parentheses

^*^ *p* < 0.05

^**^ *p* < 0.01

^***^ *p* < 0.001

**Source: Authors Calculation from LASI data**.

**Note:** Column 1–5 represents ordinary least square while column 6 represent IV regression. The dependent variable is equal to 1 if a person is working or looking for work. Health as an independent variable is defined as 1 if an individual reported poor or fair health status, otherwise, it is 0. ®-Reference category.

Apart from age, socio-economic variables also tend to play a major role in determining ill-health effect on labour force participation. In India, people residing in rural areas, socio vulnerable groups and poor people are more likely to engage in informal and unorganised sectors, thus leaving them with no employment and health insurances. Thus they may have to continue to work despite their sad state of health. As provided in Table 2 on effect of health on labour force participation, we found that people living in urban areas are more likely to leave work force than people in rural areas. People from vulnerable groups such as Schedule caste are more like to continue to work despite ill-health. While with increase in MPCE quintile we found a negative association between ill-health and labour force participation.

### Heterogeneous effect of Ill-health on labour force activity by age

Age not only signifies a biological marker but also brings along social, economic and cultural specificities and obligations in countries like India. It is, therefore, important to investigate the effect of health on work status by age. A prime aged male adult is expected to work to support his family while elderly may have to continue his work due to lack of familial support or lack of social security benefits for sheer survival. So the question arises whether behaviour during the ill-health on labour force participation differs by age group. Table 3 presents the effect of ill-health on work status with respect to middle aged and elderly individuals. In both the age groups, with the decline in health there is higher chance of decline in labour force participation. Undoubtedly, the decline is higher amongst the elderly than among the middle age adult group. The decline among middle aged adults is about 28.7 percent while in elderly it is 36 percent. But what is more interesting is the differences across socio-economic factors between these two age groups. With increase in education, there is decline in labour participation among elderly while the middle aged adults were more likely to continue to work. This might to due to the fact that with higher education, there is either sufficient saving or better social security in older ages and they can afford to quit the labour market. On the contrary, among the middle aged adult group, higher education represents higher earning and productivity and therefore, there is higher incentive to remain in workforce despite the ill-health. In contrast, studies from developed countries having higher disability benefits found that less educated are more likely to leave workforce among older people [20]. However, elderly who belong to vulnerable groups such as schedule caste and other backward class also tend to be more in labour force, while the middle aged adults they are less likely to work. When it comes to the wealth quintile, poorest are more likely to work as compared to the richest persons.

**Table 3:** Estimation results of instrumental regression estimates for labor force participation by age in india.

Labour force Activity	Middle (45–59)		Elder (60+)	
	Coeff (*β*)	SE	Coeff (*β*)	SE
**Self-reported health**	-0.287^***^	-0.035	-0.362^***^	-0.039
**Gender**				
Male®				
Female	-0.138^***^	-0.007	-0.118^***^	-0.009
**Years of Schooling**	0.001^*^	-0.001	-0.012^***^	-0.001
**Marital Status**				
Others®				
Married	-0.025^**^	-0.008	-0.005	-0.009
**Spouse Employment**	0.033^***^	-0.005	0.164^***^	-0.009
**Physical Activity**				
No®				
Yes	0.106^***^	-0.006	0.249^***^	-0.01
**Ever Smoked**				
No®				
Yes	0.010^*^	-0.005	-0.020^*^	-0.009
**Ever Consumed Alcohol**				
No®				
Yes	-0.020^***^	-0.005	-0.022^**^	-0.008
**Residence**				
Rural®				
Urban	-0.030^***^	-0.007	-0.056^***^	-0.011
**Caste**				
Schedule Tribe®				
Schedule Caste	-0.005	-0.009	0.048^***^	-0.014
Other backward class	-0.016^*^	-0.008	0.057^***^	-0.013
Others	-0.032^***^	-0.009	0.025	-0.014
**Religion**				
Hindu®				
Muslim	0.004	-0.009	0.021	-0.013
Christian	-0.001	-0.009	0.047^**^	-0.015
Other	0.021^*^	-0.01	-0.008	-0.018
**MPCE quintile**				
Poorest	0.021^*^	-0.008	0.027^*^	-0.012
Poorer	0.012	-0.008	0.025^*^	-0.011
Middle	0.007	-0.007	0.017	-0.011
Richer	0.011	-0.007	0.026^*^	-0.011
Richest®				
**Children Below 5 years**	-0.014^***^	-0.003	-0.012^**^	-0.004
**Children Between 6 and 14 years**	-0.003	-0.002	-0.015^***^	-0.003

*p < 0.05

** p < 0.01

*** p < 0.001

**Source**: Authors Calculation from LASI data. The dependent variable is equal to 1 if a person is working or looking for work. Health as an independent variable is defined as 1 if an individual reported poor or fair health status, otherwise, it is 1. ®-Reference category.

### Effect of Ill-health on wage and hours worked

From the above tables and discussion, it is clear that there does not appear to be major difference in the effect of health on labour force participation, in general, between middle aged adults and elderly despite the fact that there is considerable variation across various socio-economic groups between these two groups. A unit decrease in health results in decline of employment by 0.34 to 0.5 points. However, this is not so when we analyse the wage lost due to ill-health. Table 4 presents the effect of health on wage earned for adult age group and elderly. The decline in wage earned due to ill-health is about 11 percent overall and amongst the elderly. However, we did not find any significant decline in wage among middle age groups. Even though for young and elderly there does not appear to be any difference between how ill-health effected the probability of participation in labour activity. However, when conditioned on being employed, poor health has reduced effect on wage earned among elderly but among middle age adult it did not have any effect. Across the socio economic variables, with increase in schooling years, MPCE quantile there is an increase in wage earning across the middle and elderly age groups. In addition, people living in urban areas are more likely to earn than people in rural areas, despite ill-health.

**Table 4:** Estimates from Heckman selection model on log of wage earnings and hours worked of currently working individuals.

Wage	Wage						Hours Worked					
	Overall		Middle		Elderly		Overall		Middle		Elderly	
	Coeff (*β*)	SE	Coeff (*β*)	SE	Coeff (*β*)	SE	Coeff (*β*)	SE	Coeff (*β*)	SE	Coeff (*β*)	SE
Self-reported health	-0.117^***^	-0.013	-0.111	-0.09	-0.116^***^	-0.021	-0.032^***^	-0.009	-0.026	-0.035	-0.039^*^	-0.016
**Residence**												
Rural®												
Urban	0.335^***^	-0.015	0.346^***^	-0.095	0.307^***^	-0.026	0.142^***^	-0.01	0.143^***^	-0.037	0.138^***^	-0.019
**Caste**												
Schedule Tribe®												
Schedule Caste	0.087^***^	-0.022	0.073	-0.141	0.133^***^	-0.037	0.040^**^	-0.015	0.063	-0.055	-0.001	-0.028
Other backward class	0.017	-0.02	-0.014	-0.128	0.109^**^	-0.034	0.054^***^	-0.014	0.075	-0.05	0.019	-0.025
Others	0	-0.022	-0.019	-0.142	0.074	-0.038	0.036^*^	-0.015	0.057	-0.055	0.002	-0.028
**Religion**												
Hindu®												
Muslim	0.095^***^	-0.022	0.093	-0.143	0.080^*^	-0.037	-0.066^***^	-0.015	-0.067	-0.056	-0.054	-0.028
Christian	0.098^***^	-0.023	0.074	-0.154	0.151^***^	-0.039	-0.047^**^	-0.016	-0.046	-0.059	-0.047	-0.029
Other	0.051	-0.03	0.033	-0.196	0.09	-0.052	0.018	-0.021	0.001	-0.077	0.066	-0.039
**MPCE Quintile**												
Poorest®												
Poorer	0.082^***^	-0.019	0.106	-0.127	0.045	-0.031	0.037^**^	-0.013	0.051	-0.049	0.011	-0.023
Middle	0.165^***^	-0.019	0.183	-0.129	0.154^***^	-0.032	0.079^***^	-0.014	0.093	-0.05	0.053^*^	-0.024
Richer	0.210^***^	-0.02	0.248	-0.131	0.155^***^	-0.033	0.110^***^	-0.014	0.119^*^	-0.05	0.090^***^	-0.024
Richest	0.411^***^	-0.02	0.478^***^	-0.136	0.284^***^	-0.035	0.104^***^	-0.014	0.118^*^	-0.052	0.076^**^	-0.026
Log of Age	-0.850^***^	-0.043	0.018	-0.487	-1.121^***^	-0.129	-0.331^***^	-0.03	-0.061	-0.189	-0.583^***^	-0.096
**Sex of the respondent**												
Male®												
Female	-0.535^***^	-0.016	-0.558^***^	-0.104	-0.509^***^	-0.028	-0.072^***^	-0.011	-0.101^*^	-0.043	-0.041^*^	-0.021
Years of Schooling	0.043^***^	-0.002	0.047^***^	-0.009	0.031^***^	-0.003	-0.005^***^	-0.001	-0.004	-0.004	-0.008^***^	-0.002
Marital Status	0.050^**^	-0.018	0.003	-0.136	0.060^*^	-0.027	0.003	-0.013	-0.028	-0.052	0.034	-0.02
Physical Activity	0.002	-0.014	0.045	-0.15	0.048^*^	-0.023	0.062^***^	-0.012	-0.005	-0.085	0.046^*^	-0.022
Ever Consumed alcohol	0.032	-0.016	-0.012	-0.12	0.066^*^	-0.026	-0.062^***^	-0.011	-0.08	-0.046	-0.056^**^	-0.02
Ever Smoked	0.039^**^	-0.015	0.013	-0.121	0.029	-0.026	-0.025^*^	-0.01	0.001	-0.053	-0.003	-0.018
N	46681		24394		22287		46671		24388		22283	

Standardized beta coefficients; Standard errors in parentheses

^*^ *p* < 0.05

^**^ *p* < 0.01

^***^ *p* < 0.001

Source: Authors Calculation from LASI data

The dependent variable logarithmic of total wages earned though full time and side jobs of the respondent. The dependent variable logarithmic of hours worked through full time and side jobs of the respondent. Health as an independent variable is defined as 0 if an individual reported poor or fair health status, otherwise, it is 1. Young here is defined as individuals between 18 to 59 years of age. Elderly are defined as individuals who are 60 and above age.

Table 4 shows, the effect of ill-health on hours worked. With ill-health, there is a reduction in hours worked among elderly but this is not the case with the middle aged adults. The study did not find any significant decline in hours worked among middle aged due to ill-health. Like the estimates for ill-health, there is a substantial difference in estimates for other controls for middle aged and elderly adults. For example, for the education variable, in overall model and among elderly, we found that with increase in education there is a decline in wage earned, but we found no significant effect among middle age segment. Likewise among social groups, in overall model, as compared to Schedule Tribe, Schedule Caste, and Others are found to have higher wage earnings. When segregated by middle aged and elderly adults there was no significant difference across caste. All these suggests that as the decision to work or not (for both middle and elderly it was withdrawal from labour force with ill-health) and the decision to earn wages and the decision on how many hours to work appear to be determined by different mechanism.

### Robustness

It is often considered that the selection of instrument can have varying effect on the relationship between health and labour force participation. In addition to the endogeneity of health variable, there are alternative views on the health indicator itself particularly between subjective measure of health and objective measure of health [21, 22]. Both these measure have their respective advantages and limitations. Most of the studies use Self-rated health as subjective measure and disease or ADL as an objective measure to estimate the effect of health on labour supply [11, 21, 23]. Some studies also used self-reported health as a health measure and objective measures as an instrumental health variable as reported in Table 5. Across all the age groups we found that with decline in health there is a decline in labour force participation irrespective of health measure (here we have considered any chronic disease and difficulty in mobility). The reduction in labour force activity has varied consistently across instrumental variable and ages. When chronic morbidity is considered as an instrument, it is found that reduction of labour activity is around 0.3 for overall as well as for elderly while for middle-aged adults, it is around 0.28. When considered mobility, the self-reported health has declined 0.28 among overall as well as for middle-aged adults, while reduction is found to be much higher among elderly. Across all the models with each unit increase in age there is reduction of 1.6 percent to 0.6 percent of labour activity is observed.

**Table 5:** Instrumental regression by various health instruments.

Labour Force Activity	Chronic Morbidity		Mobility		Middle Chronic Morbidity		Elderly Chronic Morbidity		Middle Mobility		Elderly Mobility	
	Coeff (*β*)	SE	Coeff (*β*)	SE	Coeff (*β*)	SE	Coeff (*β*)	SE	Coeff (*β*)	SE	Coeff (*β*)	SE
**Self-reported health**	-0.318^***^	-0.022	-0.260^***^	-0.019	-0.282^***^	-0.024	-0.323^***^	-0.038	-0.282^***^	-0.024	-0.323^***^	-0.038
**Age at last birthday**	-0.016^***^	0	-0.016^***^	0	-0.006^***^	-0.001	-0.014^***^	-0.001	-0.006^***^	-0.001	-0.014^***^	-0.001
**Gender**												
Male®												
Female	-0.131^***^	-0.006	-0.135^***^	-0.006	-0.139^***^	-0.007	-0.120^***^	-0.009	-0.139^***^	-0.007	-0.120^***^	-0.009
**Years of Schooling**	-0.005^***^	-0.001	-0.004^***^	-0.001	0.001^*^	-0.001	-0.012^***^	-0.001	0.001^*^	-0.001	-0.012^***^	-0.001
**Marital Status**												
Others®												
Married	-0.012	-0.006	-0.011	-0.006	-0.025^**^	-0.008	-0.005	-0.008	-0.025^**^	-0.008	-0.005	-0.008
**Spouse Employment**	0.078^***^	-0.005	0.077^***^	-0.005	0.033^***^	-0.005	0.163^***^	-0.009	0.033^***^	-0.005	0.163^***^	-0.009
**Physical Activity**												
No®												
Yes	0.172^***^	-0.006	0.177^***^	-0.005	0.106^***^	-0.005	0.254^***^	-0.01	0.106^***^	-0.005	0.254^***^	-0.01
**Ever Smoked**												
No®												
Yes	-0.004	-0.005	-0.004	-0.005	0.010^*^	-0.005	-0.019^*^	-0.009	0.010^*^	-0.005	-0.019^*^	-0.009
**Ever Consumed Alcohol**												
No®												
Yes	-0.023^***^	-0.005	-0.019^***^	-0.005	-0.020^***^	-0.005	-0.019^*^	-0.008	-0.020^***^	-0.005	-0.019^*^	-0.008
**Residence**												
Rural												
Urban	-0.040^***^	-0.007	-0.037^***^	-0.006	-0.030^***^	-0.007	-0.054^***^	-0.01	-0.030^***^	-0.007	-0.054^***^	-0.01
**Caste**												
Schedule Tribe®												
Schedule Caste	0.014	-0.008	0.007	-0.008	-0.005	-0.009	0.042^**^	-0.014	-0.005	-0.009	0.042^**^	-0.014
Other backward class	0.013	-0.008	0.008	-0.007	-0.016^*^	-0.008	0.053^***^	-0.012	-0.016^*^	-0.008	0.053^***^	-0.012
Others	-0.014	-0.008	-0.021^*^	-0.008	-0.033^***^	-0.009	0.021	-0.014	-0.033^***^	-0.009	0.021	-0.014
**Religion**												
Hindu®												
Muslim	0.013	-0.008	0.01	-0.008	0.004	-0.009	0.019	-0.013	0.004	-0.009	0.019	-0.013
Christian	0.024^**^	-0.009	0.026^**^	-0.009	-0.001	-0.009	0.048^***^	-0.014	-0.001	-0.009	0.048^***^	-0.014
Other	0.007	-0.011	0.007	-0.011	0.021^*^	-0.01	-0.008	-0.017	0.021^*^	-0.01	-0.008	-0.017
**MPCE quintile**												
Poorest	0.022^**^	-0.008	0.022^**^	-0.007	0.021^**^	-0.008	0.027^*^	-0.012	0.021^**^	-0.008	0.027^*^	-0.012
Poorer	0.017^*^	-0.007	0.017^*^	-0.007	0.012	-0.008	0.025^*^	-0.011	0.012	-0.008	0.025^*^	-0.011
Middle	0.01	-0.007	0.011	-0.006	0.007	-0.007	0.018	-0.011	0.007	-0.007	0.018	-0.011
Richer	0.018^**^	-0.007	0.018^**^	-0.006	0.011	-0.007	0.026^*^	-0.011	0.011	-0.007	0.026^*^	-0.011
Richest®												
**Children Below 5 years**	-0.014^***^	-0.003	-0.014^***^	-0.003	-0.014^***^	-0.003	-0.012^**^	-0.004	-0.014^***^	-0.003	-0.012^**^	-0.004
**Children Between 6 and 14 years**	-0.012^***^	-0.002	-0.012^***^	-0.002	-0.003	-0.002	-0.015^***^	-0.003	-0.003	-0.002	-0.015^***^	-0.003
**r2**	0.234		0.263		0.026		0.154		0.026		0.154	
**F**	743.332		782.444		83.101		304.188		83.101		304.188	
**N**	46021		46021		24283		21738		24283		21738	

## Discussion

This paper tries to address the impact of ill-health on labour force outcomes by age groups in India. With the lack of credible social security measures, even elderly continues to work in India and therefore, the impact of ill-health and labour outcome are particularly important among them. This study tries to fill this gap by examining the effects of health on both labour withdrawal, hours worked and wages earned among the middle-aged and older adults. First, the effect of health on labour force withdrawal is observed among both middle aged and elderly adults. The withdrawal of labour participation is higher among elderly as compared to middle aged adults. We also found that the mechanism of the relationship will be different for elderly and adult ages across the socioeconomic groups. We found that elderly who belong to vulnerable social groups and poor economic classes are more likely to work despite ill-health while we found an opposite relation with respect to middle aged adults. The findings are contrary to earlier research [2] which might be due to people’s tendency to neglect health and prioritise work [12]. However, the overall association between ill-health and labour participation is found to be negative among both middle-aged and elderly adults in India.

Second, we found that with ill-health, there is a decline in hours worked and wages earned among elderly, while among middle aged adults there is no significant difference. This is despite the fact that ill-health has negative effect on labour participation both among middle-aged and elderly cohorts. Our findings are in contrast to what was found in developed countries. In developed countries evidence has indicated that people may still remain in labour force by adjusting working hours despite ill-health [4], or may exhibit an increase in hours worked after an acute health shock [20]. In addition, evidence from UK also suggests that effect of health on wages and hours worked is highest in people when suffered from a permanent condition and during their peak life cycle earnings [24]. Such wide variation across European countries has been captured by Trevisan & Zantomio (2016) and they have attributed the chance of retention of employment after a health shock mostly to disability benefits. However, the evidence in developing countries has different stories, when fallen ill there is a reduction in wages and hours worked in India [15, 25] and there is a loss of income in the households wages [2, 26]. The different mechanism of work when affected by ill-health with respective to different countries can be attributed to different policies, nature of work and social policies. Such absence of benefits from developing countries like India, suggests that decision to engage in work when or after suffering from a health shock is often determined by different mechanisms.

Third, results across sub-groups by age reveals that vulnerable groups are more likely to earn a lesser wage and engage in lesser work hours due to ill-health. This finding is consistent with Kumara & Samaratunge (2018) [2] for Sri Lanka and Ghatak & Madheswaran (2014) [27]. In the Indian context, both the studies found that the decline in wages is higher among vulnerable groups. Additionally, in line with our findings from an analysis on Indian rural households, a study found that short-term illnesses also have an impact on both earnings and wage employment [15].

Specifically in Indian empirical literature, the conclusion on effect of health on labour earnings are based on a particular health proxy and labour outcome. However, studies have indicated that with the change in health measure, the effect on labour outcomes may differ [21]. Therefore, considering only one health measure may provide an incomplete picture of the ground reality [12]. This study has considered all the shortcomings discussed above, and found that irrespective of the health measure as an instrument, there is a reduction in labour participation among both middle-aged and elderly. However, the magnitude has varied with the health measure.

These findings present profound implications for the labour market and health shocks linkages, especially when even among the regular waged employed in the non-agriculture sector, close to 54% of the population were not entitled to a paid leave [28]. Absence of social security nets, especially in the informal sector in India, accompanied with an excess burden of household responsibility on the middle aged population, plunges them into a doubly disadvantaged position of subpar labour market outcomes (in the form of lesser hours of work or lower wages) and unfair healthcare treatments. This effect is further amplified by the spill-overs that maybe present in a household with a sick breadwinner, thereby creating a continued cycle of impoverishment for families suffering from adverse health shocks.

A key strength of our study lies in the use of diagnosed disease as one of the proxy measures of health. Our findings rely on multiple robustness checks using different health measures and our main conclusion holds true across different health measures. However, there are some limitations to our study. Firstly, further research is needed on the understanding of labour outcomes in developing countries, especially in countries where limited access to health care may push people with undiagnosed health issues to remain in the labour market. Moreover, in countries like India, when people employed in informal sectors fall sick, it is more likely that another family member may take up a similar job. However, the present analysis does not have the scope to explore this spill over effect. Thirdly, we did not consider multi morbidity and its subsequent effect on labour outcomes, as it is likely that people suffering from more diseases may be more prone to withdraw from labour markets.
